# Editorial

**Published:** 1864-11

**Authors:** 


### Editorial.

### THE RUBBER PATENT QUESTION AGAIN.

We refer to this subject not to discuss dental patents, but to glance at the phase of the subject that has recently come up.

On the 7th of June last, John A. Cummings, a dentist of Boston, obtained a patent, in which he sets up the following claim viz: "What I claim, or desire to secure by letters patent, is, forming the plate and gums in which the teeth are set, in one piece o hard rubber or vulcanite, and making it sufficiently hard to bear mastication and yet yield a little to the motion of the mouth, or in other words, retain a portion of its elasticity as herein set forth." Here we have *Monsieur Tonsin come again.* This patent was obtained, as we understand, after three separate rejections. He first applied for a patent in May, 1855, and at that time, and twice again, his application was rejected. The last rejection was for want of novelty. He finally, however, succeeded, perhaps because of his importunity.

This patent is now menaced over the heads of the profession, with threats of prosecution to all who will not comply with the terms that may be dictated. Now we regard this as a base imposition, to say the least, on the profession.

A patent for vulcanizing rubber and other gums was granted to Goodyear in 1851 and re-issued in 1858; and with that we never felt disposed to take issue, it was a matter that originated outside of the profession, and its application to dentistry was a very small fraction of its uses. It was applicable not only to the preparation of vulcanite base for artificial teeth, but to the production of hundreds and perhaps thousands of different articles to be used for as many different purposes. We suppose the patent was properly and in good faith applied for, and granted in good faith; and we have regarded it as valid, and acted upon that principle. We have always supposed that any one having obtained a license under that patent, could apply it to the manufacture of any article he might choose, if there was no limitation.

It certainly is not admissable to take out a new patent for any or every article in the manufacture of which vulcanized rubber might be employed, and hence Cummings' rejection at one time, and justly too, "for want of novelty." It would be a most absurd thing to grant a new patent for the application of vulcanized rubber to the manufacture of each and every article from a penny whistle to an elaborate piece of furniture; and yet if the principle upon which Cummings obtained his patent is a correct one, this can be done. In our opinion, justice and common sense both forbid it. We should like very much to see how a United States Court would settle this question.

This man Cummings, doubtless, has a large income in prospect, but it is yet in an air castle; for he proposes to furnish a license to dentists, for their special use, for the very moderate tax of one dollar per piece, whether of one tooth or fourteen, and two dollars for every full set.

We are not informed whether this is to extend back to cover all the cases that have heretofore been made, or whether it is to start from the date of the patent, or from the date of the license; but we presume such an extraordinary document could reach back indefinitely. That would greatly enhance the income of the patentee, and he will perhaps remember the adage, that under extraordinary circumstances, extraordinary things may be done.

This patent was not suffered to remain in the hands of one man, but was placed in the hands of a company called the Dental Vulcanite Company, which would have a greater influence, more power and money, with which to carry out the object. This fact might suggest inherent weakness in the patent, and a want of faith in its merit.

But we are glad to know that this Company, this power, which was to overawe and over-ride the dental profession is to be met by another company, combination or power, in the shape of a "*Dental Protective Union*," who will contest its right to proceed in the work of taxation till that right is thoroughly tested and the whole matter definitely settled.

Prosecutions have already been instituted against several dentists for infringement of this patent, by the use of vulcanized rubber for artificial dentures, and of course everything possible that can be done by money and talent will be employed to insure its success.

This is a matter in which the whole profession is interested, and one concerning which every dentist should place himself in a safe position. We would suggest that every one who desires protection in this or any similar matter should at once become a member of the Dental Protective Union, which now has its headquarters in Boston, but its members all over the Union; this may be done by a contribution of $10. Every member has the protection of the Union, and in the event of a prosecution, the case is conducted and the expenses paid by it. The amount is a small matter, and should be freely contributed by every one for the common good, even though he were in no danger himself.

If these first cases are gained by the prosecutien, every member of the profession is immediately in danger, and may suffer much greater loss than if he were to contribute his mite at once. We hope this matter may be soon tested and the profession not kept in suspense as on former occasions; but it will depend much upon the promptness with which the profession respond to the call of the Protective Union. The profession should not expect two or three men to be at an expense of perhaps thousands of dollars in defending a case or cases in which all are deeply interested. Information upon the subject may be obtained of Dr. I. J. Wetherbee, of Boston, to whom contributions may also be sent, when he will return certificates of membership. T.

### GOLD WEB AND RUBBER COMBINATION.

We have for some time past been using this style of work, and it far exceeds our anticipations. It has several very obvious advantages. By its use the chief objections to rubber as a base for artificial teeth, are removed, and all the advantages retained.

The introduction of the web does not complicate the work, and requires little if any more time. The important parts, requiring skill, are the same in all styles of artificial dentures.

The first advantage resulting from this combination is strength —a very important one. It is well known to every dentist that the ordinary rubber work is often broken, especially when the process of construction has been in any respect deficient, or when the piece is put under the power of strong masticating muscles.

Perhaps this work may not, as a whole, be more frequently broken than other kinds of dentures used before it, but it breaks in a different way; while a gold denture will lose a tooth or two or have a slight fracture in the plate, a rubber plate will split in two pieces. Now this breakage is wholly obviated by the use of the web, at least we have not yet seen a piece of it broken, though we have seen many pieces of it in use, and some for a considerable time. We have put some of it under a very strong test, where every other kind of work had failed, and it is yet perfect.

Another advantage is that it is made thinner and far less clumsy than the ordinary rubber work ; it has been and still is one of the approbriums of the latter, that in order to obtain strength it must be made thick. With the web, the plate may be reduced to No. 26 or 28 of plate guage and still have sufficient strength.

The natural conformation of the palatine portion of the mouth may be very accurately retained, while with gold work this is difficult to do. It requires far less care in finishing to avoid cutting through the plate, than simple rubber, the web being a protection against any such accident.

It is a very important improvement, and one of which all dentists should avail themselves. We have used it for a considerable time, and are more and more pleased with it—have till now given no opinion, preferring to test it well before giving an expression in regard to it. For the details pertaining to it, we would refer all interested to Dr. J. A. McClelland, of Louisville Ky., or Dr. J. S. Walter & Co., of this city. T.

### THE PEOPLE'S DENTAL JOURNAL.

We have just received the October number of this valuable popular journal, and we think it an improvement upon all its predecessors, not that they were bad, but that this is very good. We wish it was in every family in the land; we are sure it would be the means of great good, stimulating the people to appreciate and take care of their teeth. We would advise our professional brethren every where to use their influence to extend its circulation. T.

### THE DENTIST'S MEMORANDUM AND ENGAGEMENT BOOK FOR 1865,

Is now ready for delivery from this office, and for sale at the Dental Depots. It is in three styles of binding, viz: with tucks, in morocco, at $1,50 per copy; in morocco, without tucks, $1,25 per copy; and in cloth, without tucks, $1,00. T.

### POPULAR ESSAYS, BY THE MICHIGAN DENTAL ASSOCIATION.

We have just received a copy of this little work and think it just the thing to accomplish the object sought. It is well written and to the point, and neatly gotten up, and will be the means of great good if properly distributed. No dentist has ever yet entered upon a systematic method of giving information to the people in regard to their teeth, without producing great benefit to them and good to the profession, and advantage to himself. We regard it as very important that every society in the country should enter upon this work. T.

### PERSONAL.

We see by a Syracuse paper that Dr. S. B. Palmer, recently of Tully, N. Y., has associated himself professionally with Dr. A. Westcott, who is of world-wide reputation as a dentist. We think this a change that Dr. P.'s abilities fully warranted him in making, and we doubt not it will result much to the advantage of all concerned.

Dr. W. H. Shadoan has removed to, and established an office in Louisville, Ky., where we hope and feel well assured he will meet with the success his abilities merit. T.

### NOTICE OF MEETINGS.

MUSCATINE, IOWA, Nov. 25, 1864.

DR. ——, *Dear Sir :*—It has been thought best to change the time of holding the next meeting of the Iowa State Dental Society, from the second Wednesday, (the regular appointment) to the first Wednesday in January.

The meeting will therefore take place at the city of Des Moines, beginning Wednesday, Jan. 4th, 1865, at 9 o'clock, A. M.

Our meeting in August was unusually interesting and profitable, and was well attended; but there are still a great many Dentists in our State who have not as yet manifested as much interest in the advancement of our profession as is desirable. Such, are especially invited to be present at our coming meeting. You will find that coming in contact with others in the profession, if it does not practically benefit you, it will at *least* greatly enliven your ambition to attain to the hightest objects of your chosen profession.

In addition to the regular discussion of subjects, there will be clinical operations performed before the Society by some of its members.

All are invited to bring cases of especial interest in private practice; and write essays in any branch of the profession, and present them before the Society.

Let every Dentist in the State make up his mind to continue his holidays, and attend this meeting *without fail.*

Officers of the Society are to be chosen for the ensuing year.

I am Yours, Fraternally,

WM. O. KULP, Cor. Sec'y of I. S. D. S.

---

LIST of subjects and essayists for the January meeting of the Iowa State Dental Society:

Dental Therapeutics.—Dr. Chase.
Treatment of Diseased Gums.— ——.
Correcting Irregularities.—Dr. Magill.
Extracting Teeth.—Dr. Corelson.
Sensative Dentine.—Dr. Sayles.
Facial Neuralgia.—Dr. Bronson.
Use of the File.—Dr. Trowbridge.
Pain in Dental Operations.—Dr. Robertson.

---

THE following was adopted Sept. 28, by the SOCIETY OF DENTAL SURGEONS of the city of New York, of which deceased was a member:

*Whereas*, It has pleased God to remove from our midst our most estimable friend, and professional brother, Frederick Dieffenbach and

*Whereas*, The deceased by his modest and unassuming manners, his amiable disposition, and his faithful efforts to secure that higher professional education, now recognized as essential to the competent Dentist, has greatly endeared himself to us, therefore

*Resolved*, That we hereby express our great regret at this sad bereavement, that we tender our sympathy to the family of the deceased with a copy of this resolution and that copies be sent to the *Cosmos* and *Dental Register* for publication.

WM. B. HURD, *President.*

CHAS. D. ALLDN, *Sccretary.*

